# Synthesis of computer simulation and machine learning for achieving the best material properties of filled rubber

**DOI:** 10.1038/s41598-020-75038-0

**Published:** 2020-10-22

**Authors:** Takashi Kojima, Takashi Washio, Satoshi Hara, Masataka Koishi

**Affiliations:** 1 Research and Advanced Development Division, The Yokohama Rubber Co., Ltd., 2-1 Oiwake, Hiratsuka,, Kanagawa, 254-8601 Japan; 2 Department of Reasoning for Intelligence, The Institute of Scientific and Industrial Research, Osaka University, 8-1, Mihogaoka, Ibarakishi, Osaka 567-0047 Japan

**Keywords:** Soft materials, Nanoscale materials, Nanoparticles, Structural materials, Composites, Mechanical properties

## Abstract

Molecular dynamics (MD) simulation is used to analyze the mechanical properties of polymerized and nanoscale filled rubber. Unfortunately, the computation time for a simulation can require several months’ computing power, because the interactions of thousands of filler particles must be calculated. To alleviate this problem, we introduce a surrogate convolutional neural network model to achieve faster and more accurate predictions. The major difficulty when employing machine-learning-based surrogate models is the shortage of training data, contributing to the huge simulation costs. To derive a highly accurate surrogate model using only a small amount of training data, we increase the number of training instances by dividing the large-scale simulation results into 3D images of middle-scale filler morphologies and corresponding regional stresses. The images include fringe regions to reflect the influence of the filler constituents outside the core regions. The resultant surrogate model provides higher prediction accuracy than that trained only by images of the entire region. Afterwards, we extract the fillers that dominate the mechanical properties using the surrogate model and we confirm their validity using MD.

## Introduction

Manufacturers are extremely interested in the properties of the filled-rubber^1^ constituents of tires, because they are directly related to tire performances, such as rolling resistance, wear, and wet traction. The filled rubber is a composite material made of polymers and fine filler nanoscale particles. Many researchers have applied X-ray scattering, electronic microscopy, and atomic force microscopy to examine the strong dependencies among the mechanical properties of filler morphologies^2–9^. However, it has been difficult to determine the origins of the mechanisms of the discrete mechanical behaviors, because the observations and experiments cannot clearly connect the structures with their effects.

Computer simulations can visualize the inside of a tire, and can be used as virtual experiments for tire design to improve performance^10^. Currently, coarse-grained molecular dynamics (CGMD) simulation of polymer materials are performed to analyze the relationship between the nanometer-scale structures observed by experiments and their meter-scale mechanical properties^4,11–16^. However, it is difficult to perform parametric studies on filler morphologies, because several months of computation are required to carry out a large-scale CGMD simulation that include thousands of filler particles^17^. To the best of our knowledge, comparisons of two large-scale CGMDs have been reported thus far^17,18^. From these, the cost of simulation has been a major barrier against gaining new insights to balance material properties (e.g., frequency dependencies, elastic moduli, and hysteresis).

Recently, machine learning (ML) has received a great deal of attention both in industry and academia^19,20^. ML’s application to self-driving cars is the one of the most famous examples^21^. ML has also been applied to material design^22–26^. This application is called “materials informatics”^27,28^ and is used to predict the results of future experimental trials based on past results^29,30^. The combination of ML and computer simulation, an essential tool for the research and development process, has been investigated in certain fields of computational science^31–41^. Acceleration of computer simulations has been achieved by applying ML to both enhance, and sometimes entirely replace, the simulation algorithms^42,43^. To provide faster, more accurate predictions^44,45^. Therefore, ML can reduce the simulation costs of large-scale CGMDs, making it possible to perform long-desired parametric studies of nanometer-scale structure, i.e., filler morphology, filler size, and filler types, to efficiency research of mechanical algorithms. In this paper, we introduce a surrogate large-scale CGMD model focusing on filler morphology, a lot of studies showed the strong dependencies among the mechanical properties^2–9,17,18^, to provide fast and accurate predictions as our first research. The surrogate model is established by a convolutional neural network (CNN) that treats the kinetic information of filler morphology as input data. The major difficulty with employing an ML-based surrogate model is the shortage of training data, owing to the huge computational cost required to create these data. To overcome these technical difficulties, we pursue the following two objectives:

1. A highly accurate surrogate model that succeeds with only a small number of large-scale CGMD results.
2. New insights into the relationship between nanometer-scale filler morphology and its material properties using the surrogate model.

### Problem

Tire manufacturers must balance tire performance against the mechanical properties of the nanometer-scale filler morphology and the resultant effects. For example, to improve wear resistance, a high elastic modulus of the tire tread where contacts the road is preferred. Although, according to finite element method based studies, the strain of most tire tread does not reach 0.05 during rolling, tread deformation can locally exceed a strain of 0.3, such as at the bottom of a groove^46,47^. Therefore, in this study, stress at a strain of 0.3 under uniaxial deformation is regarded as the predictive property.

## Results and discussion

### CNN-based surrogate model trained by the entire large-scale region data

#### Training data

**Figure 1:**
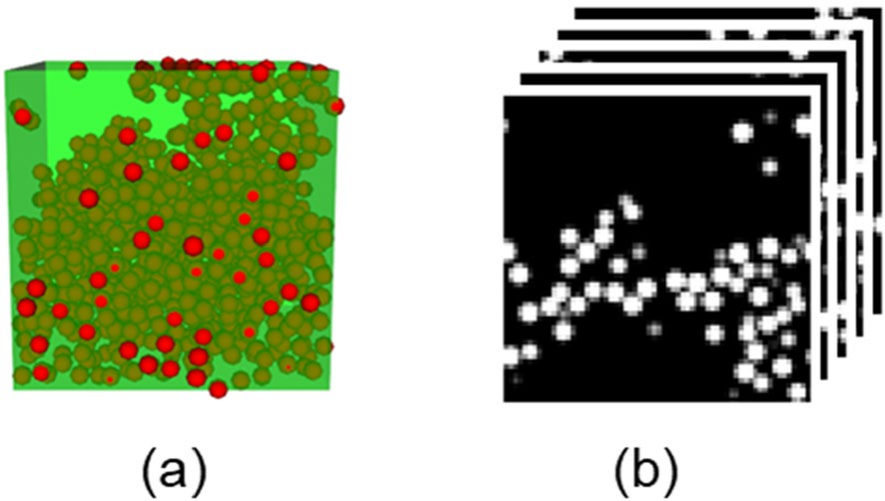
Example of a filler configuration and 3D image: (**a**) Visualized filler configuration and (**b**) 3D image.

We established a surrogate model trained by a set of 3D images representing 32 × 32 × 32-pixel 3D filler morphologies and stresses resulting from the large-scale CGMD. The training data consist of images of the filler morphologies at a strain of 0.0 and stress values at a strain of 0.3. The binary image was created using the filler density distribution. We created two images from one filler morphology, because Z was the deformation direction. One 3D image was stacked the XZ plane images along the Y direction. The other was stacked the YZ plane images along the X direction. Mirror images of X, Y, and Z directions were shifted half-a-cell or half-a-pixel toward each direction. Via the data augmentation process, 256 images were created from one filler morphology. From 45 filler morphologies, 11,520 datasets were prepared. Figure 1 shows the visualized filler configuration and the binary image of one filler morphology.

#### Training and prediction accuracy

**Figure 2:**
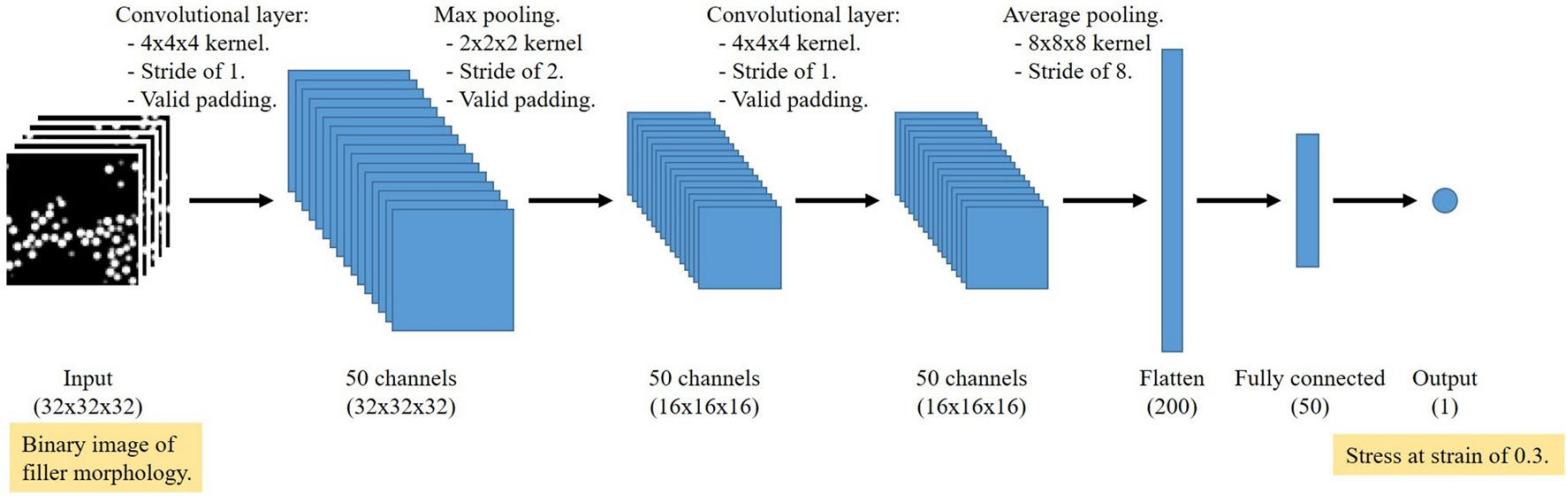
CNN architecture.

We used PyTorch to construct a 3D-CNN with two convolutional layers and one fully connected layer. The number of features of both convolutional layers was 50. The kernel of size of 4 × 4 × 4 moved with a stride of 1 in both layers. The pooling size of the first convolutional layer was 2, and global average pooling^48^ was used to connect the second convolutional layer to the fully connected layer. A leaky rectified linear unit (leaky ReLU^49^) was used as the activation function to avoid zeroing signals during training. Adam^50^ was used as the optimization algorithm. During training, 25% of the CNN was eliminated via dropout^51^. The learning rate and batch size were 0.0025 and 50, respectively. Our network architecture is shown in Fig. 2.

The datasets were divided into 10 data groups, which were separated into three instances: training, validation, and prediction. One data group was used as the validation data, and another was used as the prediction data. The remaining eight were used as the training data. The CNN was trained for 60 epochs, and the prediction accuracy was measured every 2 epochs by applying the validation data not used for training. The highest-accuracy model was selected based on the history of the prediction accuracy. The stresses of the prediction data were then calculated using the model. Changing the allocation of datasets, the stresses of all filler morphology were calculated.

**Figure 3:**
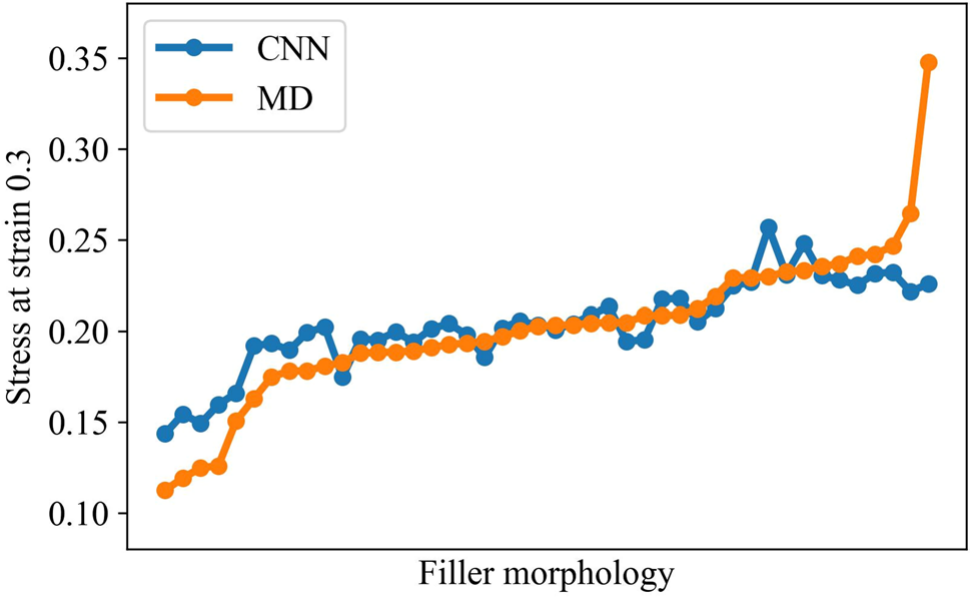
Values predicted by the CNN-based surrogate model and correct results of MD.

Figure 3 presents the correct values provided by CGMDs and predicted values via CNN. The filler morphologies of X axis were sorted by the stress provided by CGMDs at a strain of 0.3. Furthermore, we confirmed that the prediction accuracies of filler morphologies whose stresses were close to the mean value, a stress of 0.2, was high. However, the prediction accuracy corresponding to the maximal or minimal stress values was lower. We speculate that the extrapolation prediction caused this prediction accuracy to decline because the training data for prediction only included stresses less than maximal.

### CNN-based surrogate model trained by the divided middle-scale region data

#### Data argumentation by dividing the entire region

**Figure 4:**
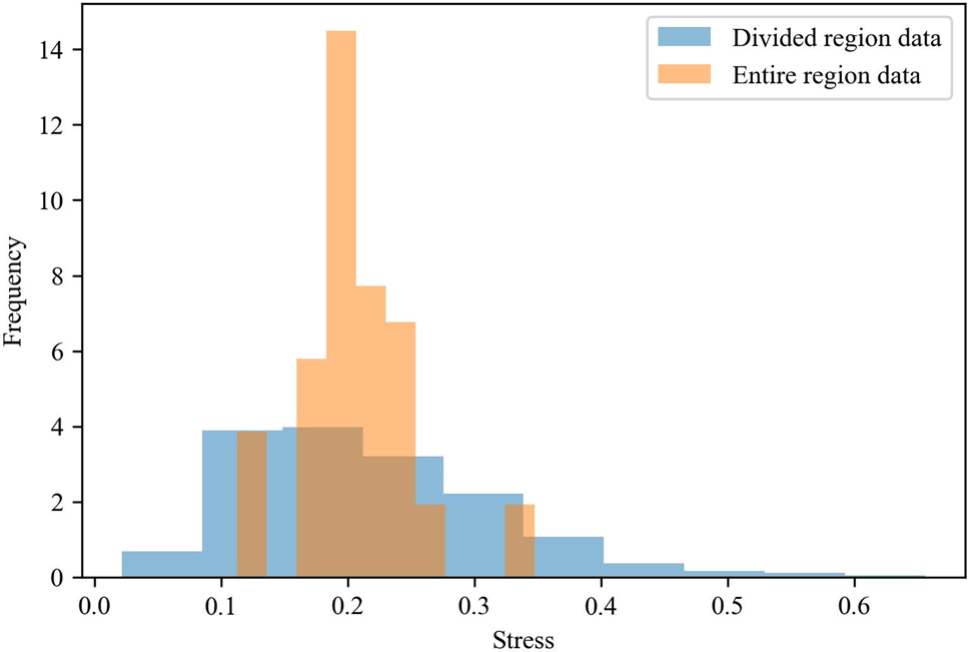
Distribution of stress in the training data.

In the previous paragraph, the entire region of the large-scale CGMD model was converted into training data. Even when 256 images were generated per morphology, the corresponding stresses reflected the same value. Although the number of input image patterns increased, the number of output stress patterns did not. To alleviate this problem, we increased the number of instances in the training data by dividing the simulation results of an entire large-scale geometrical region into middle-scale regions, a portion of the large-scale region. Each training instance was then converted into a 3D image of the middle-scale filler morphology and stresses at a strain value of 0.3 of the corresponding region. The stress of each divided middle-scale region was calculated based on the CGMD simulation algorithm, defined as $\sigma_{z} - \left( {\sigma_{x} + \sigma_{y} } \right)/2$, where the elongation direction is z. Here, $\sigma$ represents the stress of each direction, subscript the direction. The beads and bonds, in the middle-scale region, were extracted under the periodic condition. Then, the stress of each direction was calculated as the summation of the interaction force between two beads, the bond force, and the pressure arising from bead velocity. The image that was used included the fringe, in addition to its own region, to reflect the influence of the filler particles outside the divided region. This was the size effect of the large-scale CGMD. The CNN-based surrogate model was trained using these datasets. The 3D images of the divided regions were created by stacking the XZ-plane images in the Y direction and the YZ plane images in the X direction. The middle-scale instances that were used for training were sampled at 5 × 5 × 5 = 125 points, that is, 5 points along the X, Y, and Z directions were sampled. 8 mirror images were also created. Thus, 90,000 (125samples per a large-scale model × 8 mirror images × 2 types of images × 45 large-scale models) training instances of the middle-scale regions were generated from the 45 large-scale CGMD results. Figure 4 shows the distribution of the stresses of the entire region and those of the divided region. The stress of the divided region was observed to be more widely distributed than that of the entire region.

### Influence of fringe size on accuracy

**Figure 5:**
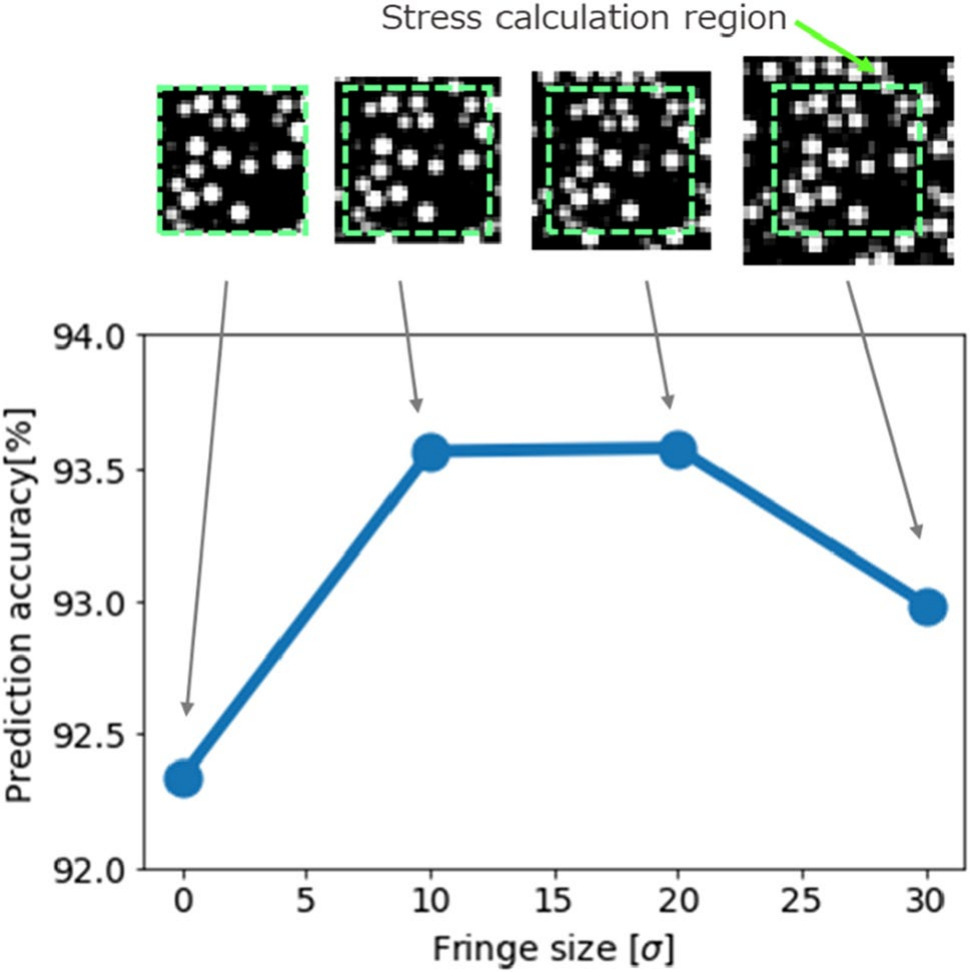
Relationship between fringe size and prediction accuracy.

We explain the relationship between the size of the fringe and prediction accuracy. The volume of the divided region was 1/8 of the entire region, and the ratio of the cell lengths of X, Y, and Z directions was 1/2. The top five morphologies, ranked by their stress magnitude, were used for the prediction. This was intended to lead to an increase in the prediction accuracy involving large stress. The data of 40 morphologies, except for these five, were used as training or validation data. The most accurate surrogate model tested by the validation data in the course of 60 training epochs was used for prediction. The averages of the prediction accuracies of the 5 morphologies having fringe sizes of 0, 10, 20, and 30[σ] were compared. The size of the entire region was 300[σ], and the number of pixels along each direction was 32. The stresses of the entire regions, calculated as the average of the divided regions included in the corresponding entire region were compared with the correct answer (i.e., the result of CGMD). The prediction accuracy of the fringe sizes of 10 or 20[σ] was similarly high, as presented in Fig. 5. Therefore, 10[σ] was selected as the fringe size because of its smaller cost for image creation.

**Figure 6:**
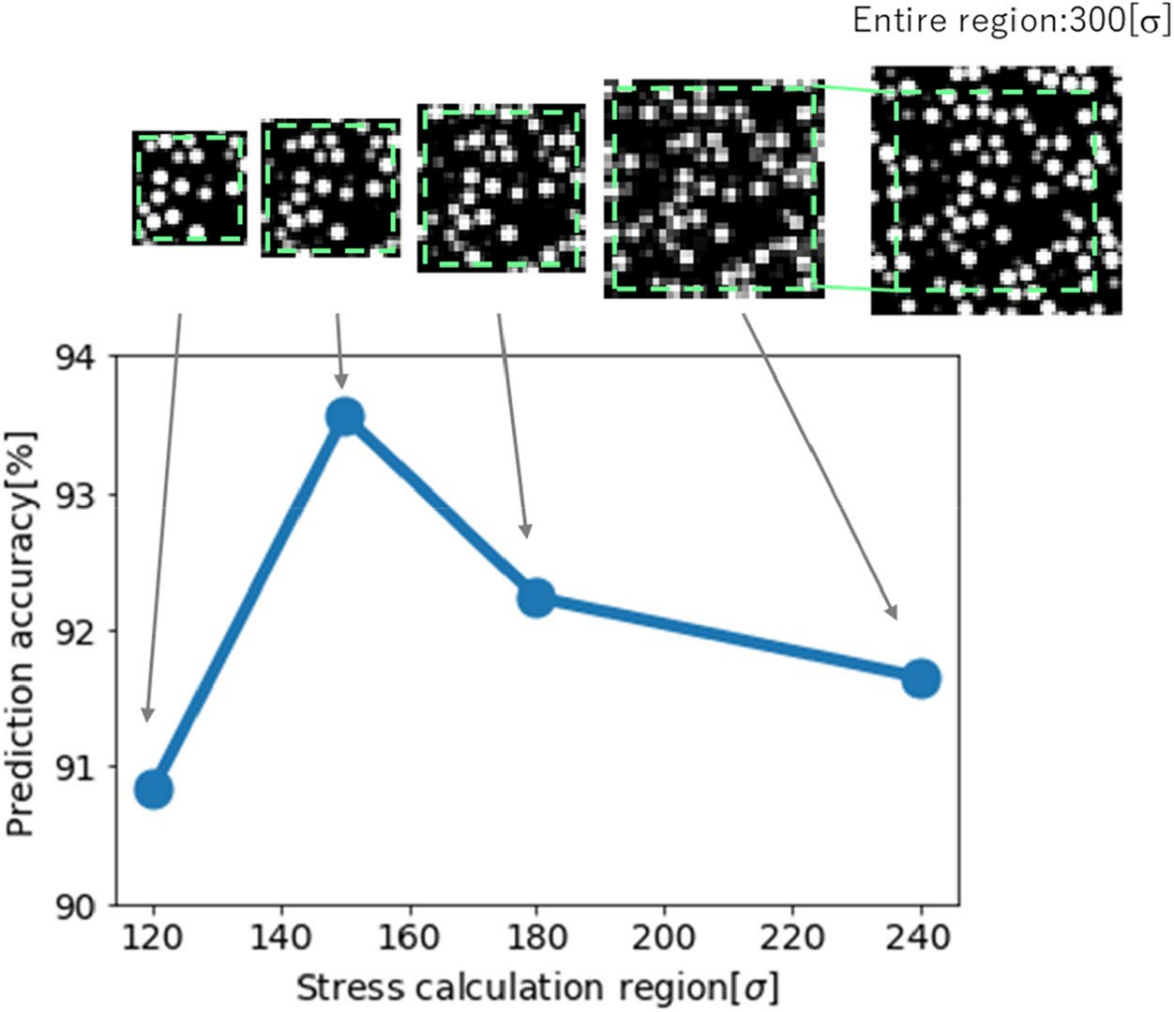
Relationship between the middle-scale region and the prediction accuracy. Here, the fringe size was 10[σ].

Next, we explain the relationship between the size of the divided region and the corresponding accuracy. The accuracies of the divided region sizes of 120, 150, 180, and 240[σ] (i.e., 40, 50, 60, and 80% of the entire region, respectively) were measured. Note that the size of the fringe was 10[σ] based on the result of a previous paragraph. Figure 6 shows that 150[σ] indicated the highest accuracy. We deduced that the distribution of the stress of the larger divided region was smaller than that of the smaller divided region because the stress of the larger divided region was close to that of the entire region. As a result, the distribution of the training data of the divided region was narrower and similar to that of the entire region’s training dataset. Thus, the accuracy decreased. On the other hand, when the divided region is smaller, the stress distribution is wider. However, the accuracy still declined, owing to other effects from increasing factors that were not represented in the binary images (e.g., crosslinking of polymer). Thus, we set the stress calculation region in the divided region to 150 × 150 × 150 [σ], and the imaging region to 170 × 170 × 170 [σ] by adding a fringe of size of 10 [σ] to the stress calculation region.

### Prediction accuracy of the surrogate model trained by the divided region

**Figure 7:**
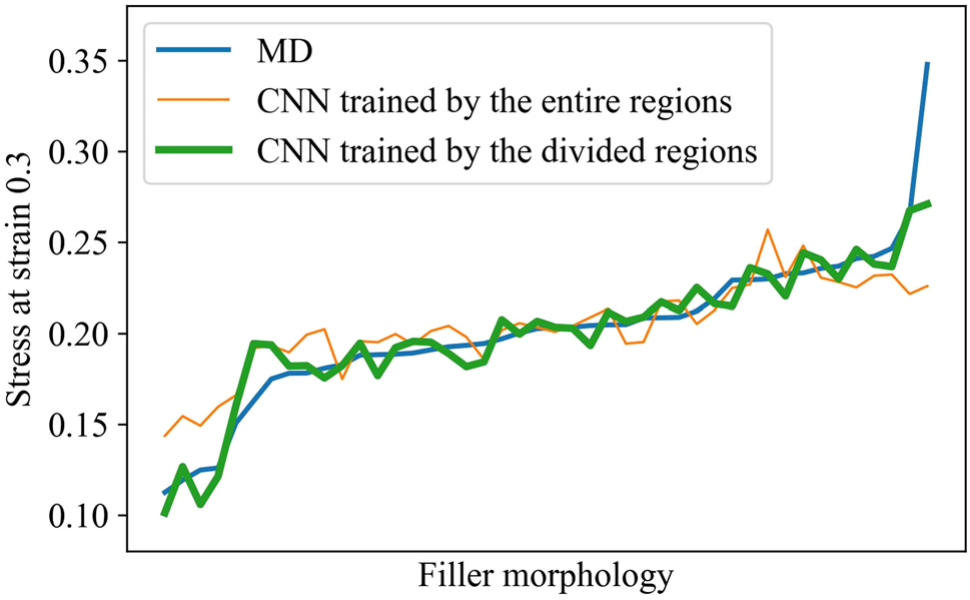
Prediction accuracy of the surrogate model trained by the divided region data.

We established the surrogate model for the divided region under the conditions described in the previous section. The configuration of the CNN and training condition were the same as those used for the entire region. Figure 7 shows the accuracy of the CNN-based surrogate model trained by the divided regions. The model trained with the entire region showed lower prediction accuracy for minimal and maximal stress because of the extrapolation prediction. In contrast, the prediction accuracy increased for the model trained with the divided region data. This improvement resulted from the wider distribution of the stress data used for training. Thus, it can be inferred that some divided regions in the maximal stress morphology were predicted via interpolation.

### Mechanism of the mechanical behavior of the filled rubber

#### Extraction of highly contributing filler particles

We calculated the contribution of each filler particle using the surrogate model to study the mechanism of the mechanical behavior. The contribution, *C(x*_*n*_*)*, of a filler particle, *x*_*n*_, reflects the amount of change in the stress caused by the presence or absence of the particle. This is given by Eq. (1):

1 $$\begin{aligned} C\left( {x_{n} } \right) & = f\left( {X|x_{1} ,x_{2} , \ldots ,x_{n - 1} ,x_{n} ,x_{n + 1} , \ldots ,x_{999} ,x_{1000} } \right) \\ & \quad - f\left( {X|x_{1} ,x_{2} , \ldots ,x_{n - 1} ,x_{n + 1} , \ldots ,x_{999} ,x_{1000} } \right), \\ \end{aligned}$$

where X and *f(X)* stand for the filler morphology and the function that calculate the stress from the filler morphology, X, respectively.

**Figure 8:**
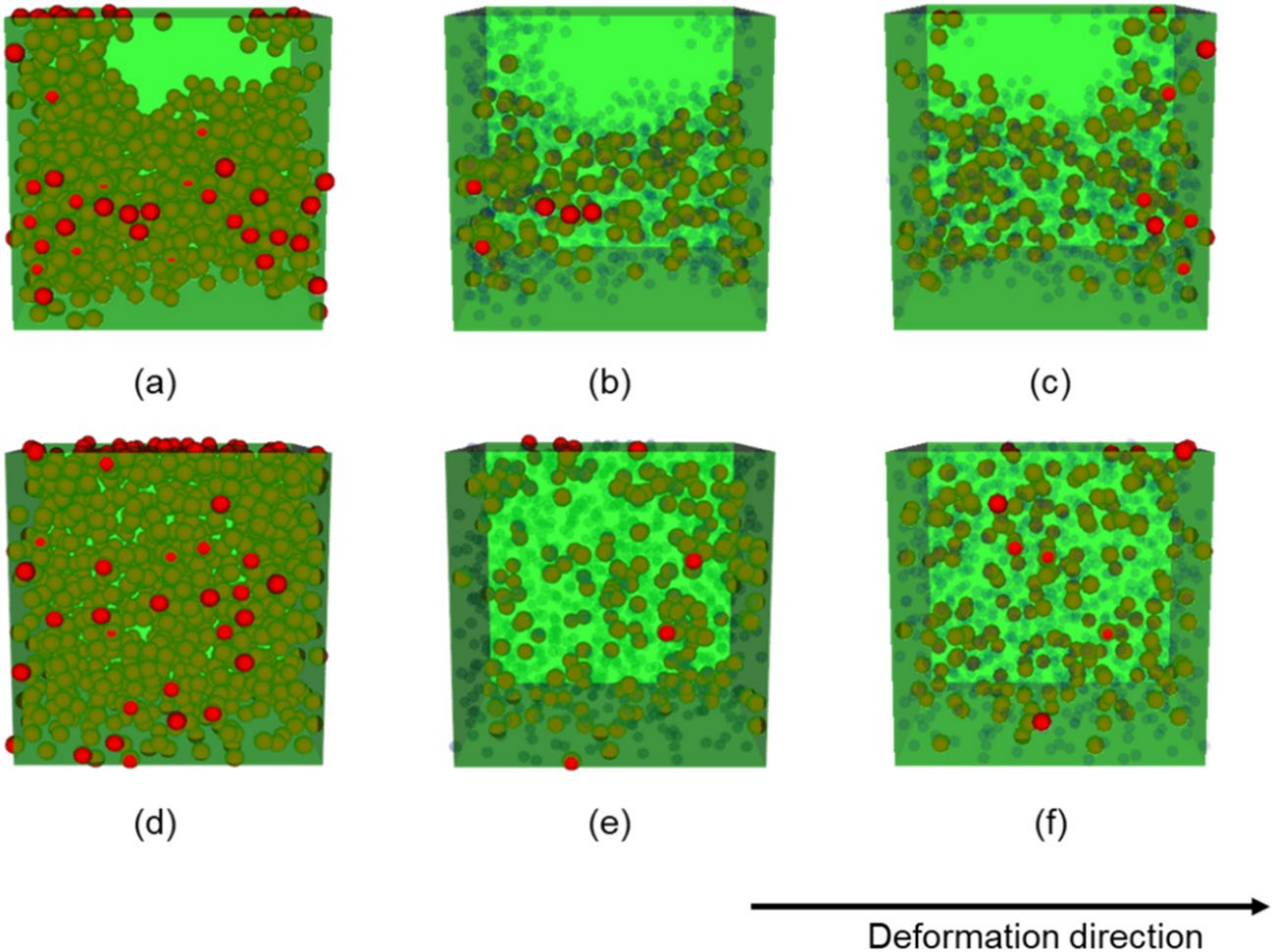
Filler configurations of the extracted highly contributed fillers and randomly selected filler. The green box is the entire region of the large-scale CGMD model. The red particles are the extracted fillers. The blue points are the non-extracted fillers. (**a**)–(**c**) Reflect the maximal stress morphology. (**a**) Includes all filler particles, (**b**) includes the extracted 200 filler particles as the high-contributing filler particles, and (**c**) includes the randomly selected 200 filler particles. (**d**)–(**f**) reflect the minimal stress morphology. (**d**) Includes all filler particles, (**e**) includes the extracted 200 filler particles as the high-contributing filler particles, and (**f**) includes the randomly selected 200 filler particles.

**Figure 9:**
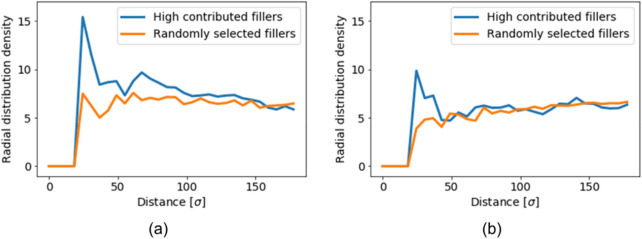
Radial distribution density functions of the extracted 200 fillers: (**a**) is the maximum stress morphology, and (**b**) is the minimal stress morphology.

Figure 8 shows the 200 filler particles that provide a large contribution to the maximal stress morphology and to the minimal stress morphology. Furthermore, randomly selected 200 filler particles are shown. The extracted filler particles were the high-contributing filler (HCF) particles in both morphologies, and aggregated compared with the randomly selected filler (RSF) particles. Moreover, the HCF particles of the maximal stress morphology were clearly more aggregated than those of the minimal stress morphology. The radial distribution density functions (RDF) shown in Fig. 9 also demonstrate this result. There is a difference between the RDFs of HCF and RSF in the minimal stress morphology at distances of 50[σ] or less. This difference implies that small aggregated structures comprising a few filler particles were extracted by our method, and the distribution of the small aggregate was similar to that of the RSF. Conversely, there was a clear difference in the maximal stress morphology at distances of 50[σ] or more. Thus, larger aggregate structures were extracted. These primary filler particles, which dominate the mechanical properties, lay parallel to the deformation direction. In summary, the size and direction of the aggregate dominated the mechanical properties.

Note that approximately 3 days were required to carry out the CGMD up to a strain of 0.3 with 1000 central processing units. Thus, using CGMD, 3000 days were needed to measure the contributions of 1000 filler particles, because 1000 CGMDs must be performed. However, our job took 2 h, including imaging, to measure the contributions of 1000 fillers using the CNN-based surrogate model. Therefore, the proposed method is 36,000 times faster than the CGMD-based method. Even considering the data creation cost using CGMD and training cost by using the data, which are 135 days and 3 h, respectively, the proposed method is 20 times faster.

### Verification with CGMD

**Figure 10:**
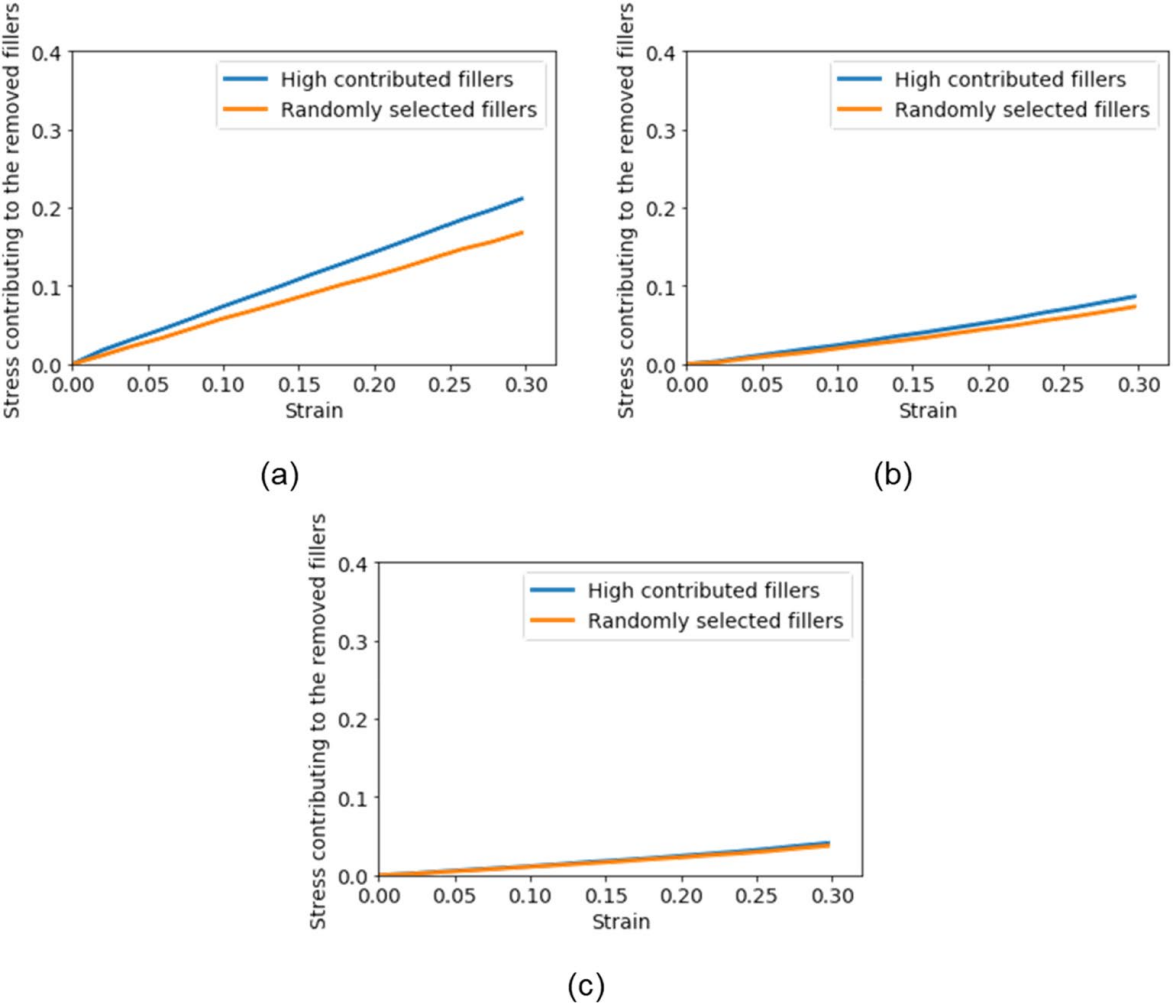
Stress–strain curves of the extracted fillers: (**a**) maximal stress morphology, (**b**) median stress morphology, and (**c**) minimal stress morphology.

The contributions of 200 filler particles extracted by the proposed method and randomly selected via HCF and RSF were calculated to verify the results of the previous section. HCF extracted from the maximal or minimal stress morphology as well as from the morphology that provided the median stress (approximately 0.2). Our previous study demonstrated that the simulation result carried out under the condition in which the interaction between filler and polymer chains was repulsive was nearly the same as that of the pure-rubber simulation^52^. Pure rubber is not added to filler particles in the polymer domain. This result implies that the repulsive interaction between filler and polymer invalidated the existence of the filler particles, although there were filler particles in the polymer melt system. Thus, the interactions between 200 filler particles, HCF and RSF, and polymer changed from attractive to repulsive to virtually remove HCF and RSF. The differences between the responses of the CGMDs, in which the interactions were attractive or repulsive, were regarded as contributions of HCF or RSF. Figure 10 presents the stress–strain curves of both conditions. Here, the simulation condition was the same as that of the previous section.

The stresses of the HCF were larger than those of the RSF were in all morphologies. The difference in responses was proportional to the stress of the system in which all 1000 fillers were validated. This result suggests that the CNN-based surrogate model succeeded in analyzing the filler morphology and measuring the filler contribution. The small difference in the minimal stress morphology implies that the contributions of all filler particles were considerably similar. The contribution of the small aggregate that comprised a few filler particles was significantly small. Meanwhile, the difference in the maximal stress morphology was large, and part of the filler morphology dominated the mechanical behavior. Summarizing these results, the large aggregate parallel to the deformation direction significantly increased the modulus of the system.

## Method

**Figure 11:**
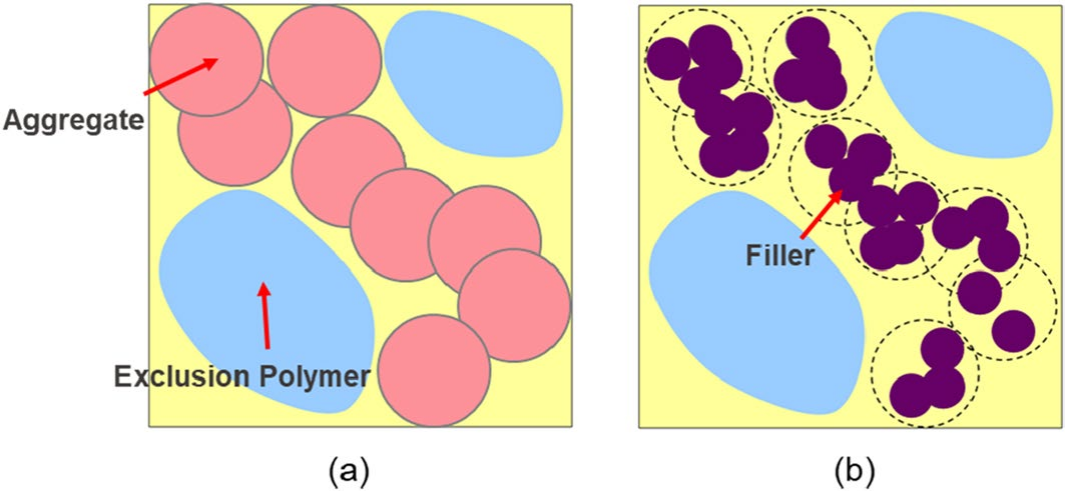
Methodology of determining the filler configuration: (**a**) 1st step; (**b**) 2nd step. In the 1st step, aggregate regions where filler particles are able to be placed and exclusion polymers where the filler particles are not placed are designed. In the following 2nd step, specified number of filler particles are distributed in the aggregate regions.

Filler morphologies are determined using multi-step Poisson point processes such that aggregated structures can be distributed effectively^53^ (Fig. 11).

First, both aggregate regions and exclusion polymers are designated based on input parameters of radius, $R_{a}$, Poisson point process intensity,$\theta_{a}$, for the aggregate region and that for the exclusion polymer,$\theta_{e}$. The number of the aggregate region, $N_{a}$, and that of exclusion polymers, $N_{e}$, are determined via Poisson point distributions of the expected values for each regions. The expected values for the aggregate region $\lambda_{a}$, and that for the exclusion polymers $\lambda_{e}$ are given by:

$$\lambda_{a} = V \times \theta_{a} ,$$

2 $$\lambda_{e} = V \times \theta_{e} ,$$

where *V* is the volume of the model. The shape of both regions is spherical. The radius of the aggregate region is given by the input parameter. The radius of the exclusion polymer,$R_{e}$, is given as follows:

3 $$R_{e} = \left( {\frac{{ - 3 \times log\left( {1 - V_{e} } \right)}}{{4 \times \pi \times \theta_{e} }}} \right)^{\frac{1}{3}} ,$$

where $V_{e}$ is the volume fraction of the exclusion polymer.

**Figure 12:**
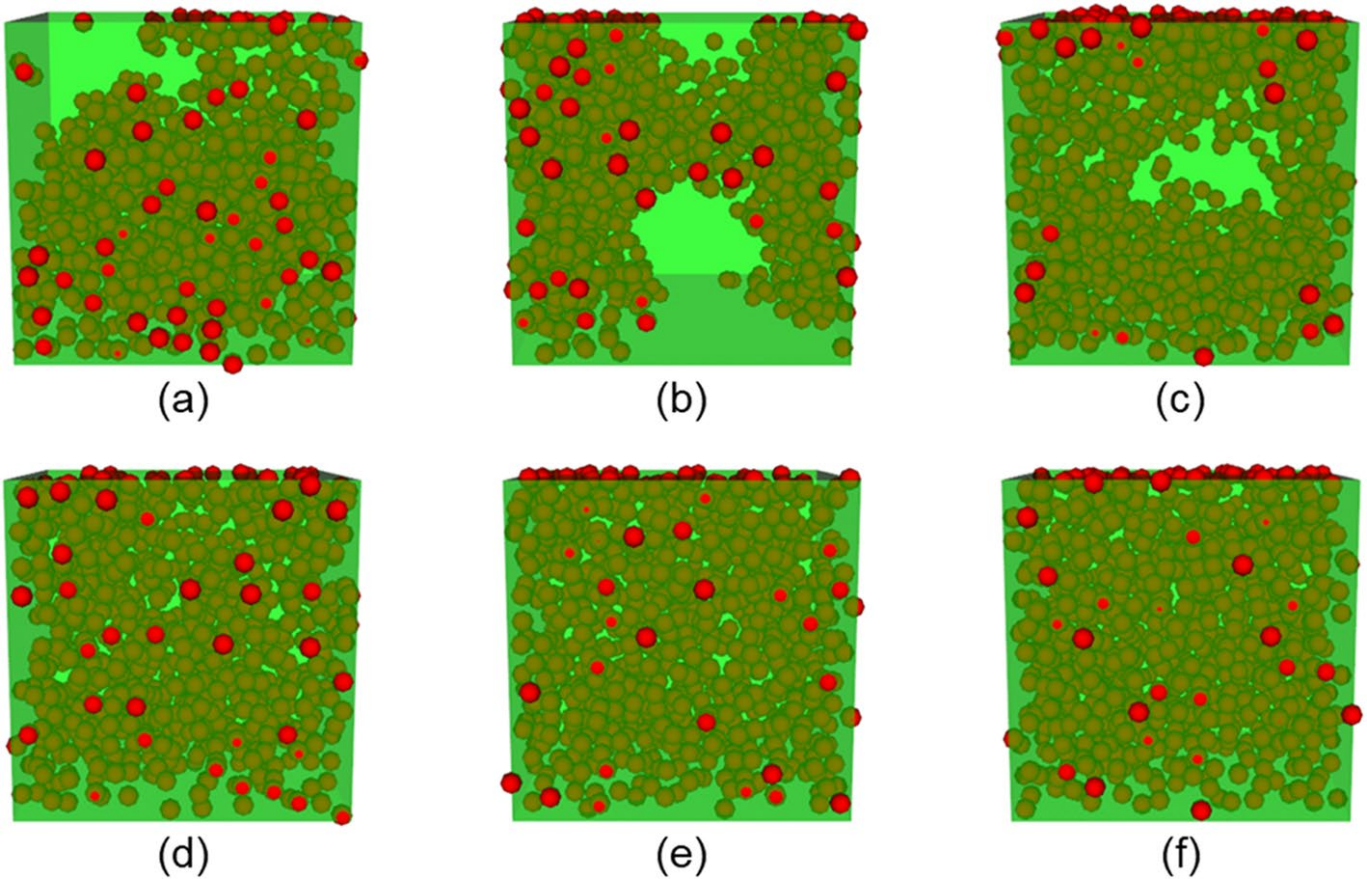
Filler configurations. The radius and the Poisson point process intensity of the aggregate, the volume fraction, and the Poisson point process intensity of the exclusion polymer are (**a**) 70,0.3,0.3,0.1, (**b**) 70,0.6,0.5,0.1, (**c**) 80,0.6,0.5,0.1, (**d**) 100,1.0,0.1,1.0, (**e**) 150,1.0,0.1,1.0, and (**f**) 200,2.0,0.1,1.0, respectively.

Second, the specified number of filler particles are placed in the aggregate region based on the two input parameters; volume fraction and filler-particle radii. In this study, the radius of filler particles is 10[σ]; where, σ is the CGMD unit of length. Although the minimum distance between the centers of the filler particles should be 20[σ] to prevent them from overlapping, we apply a minimal distance of 18[σ], because the smaller the volume fraction of the aggregate region, the lower the probability of model success. Thus, our filler particles overlap, and the configuration obtained by our multi-step process uses the molecular dynamics simulation algorithm to eliminate the overlaps. Forty-five filler morphologies are created within the range of the parameters in the multi-step process described in Table 1. Figure 12 shows 6 of the 45 morphologies. Here, the cell length and number of filler particles are 300[σ] and 1,000, respectively. The volume fraction of filler particles of all models is 15[%].

**Table 1:** Parameter range for filler configurations.

	Minimum	Maximum
Radius of the aggregate [σ]	70	200
Poisson point process intensity for the aggregate	0.3	2.0
Volume fraction of the exclusion polymer	0.1	0.3
Poisson point process intensity for the exclusion polymer	0.1	1.0

Each filler configuration is merged with 1,000 polymer chains of 20,000 bead lengths modeled using the Kermer–Grest method^54^. The polymer beads are distributed at a density of 0.85[m/σ^3^] in the system, where m is the CGMD unit of mass. By conducting reaction simulations after the merging of polymer melt and fillers, 60,000 cross-linking bonds are created. The detailed modeling techniques and simulation conditions (e.g., the interaction between filler and polymer and elongation velocity) are same as those of our previous report^18^. The stress–strain responses up to a strain of 0.3 are simulated using a molecular dynamics simulation software, LAMMPS^55^.

## Conclusion

We introduced a surrogate model of a large-scale CGMD with the aim of rapid and accurate prediction of the mechanical properties of polymerized and nanoscale filled rubber. The surrogate model was established using a CNN, the input and output data of the surrogate model were 3D binary images of the filler configuration, and the stress reflected a strain of 0.3. We increased the number of instances in the training data by dividing the simulation results on an entire large-scale geometrical region into those of middle-scale regions. Subsequently, 90,000 training instances of the middle-scale regions were generated from the 45 large-scale CGMD results. The resultant surrogate model trained on images of the middle-scale regions provided higher prediction accuracy for material elongation than that provided by the surrogate model trained using images of the entire large-scale region.

Using the higher prediction accuracy surrogate model, we developed a method that could extract the primary filler particles dominating the mechanical behavior. The large aggregates parallel to the deformation direction were extracted from the maximal stress morphology, and the small aggregates were extracted from the minimal stress morphology. These filler aggregates suggested that the large aggregate parallel to the deformation direction was key to controlling the mechanical properties.

We ran the large-scale CGMD simulation to confirm the influence of the extracted filler particles as predicted via the ML-based surrogate model. The contributions of those fillers were larger than those of the randomly selected fillers. These results led to the conclusion that the ML method could successfully predict the relationship between filler morphology and mechanical behavior. Thus, we can now predict the response of a large-scale region by integrating the responses of the middle scales. Furthermore, we find that the large aggregate that is parallel to the deformation direction is key to controlling the mechanical properties of the filled rubber. Moreover, we are considering ways to increase the prediction accuracy and expand the surrogate model to account for both filler size and the interaction energy between various polymer and filler types. In our future research, we seek to perform a parametric study of nanometer-scale structure, especially filler morphology, filler size, and interaction between filler and polymer modeling the filler types, to obtain better material properties using the established surrogate model reported herein.
